# An effector–reporter system to study cellular signal transduction in strawberry fruit (*Fragaria ananassa*)

**DOI:** 10.1038/s41438-021-00493-3

**Published:** 2021-03-10

**Authors:** Baozhen Zeng, Tianyu Li, Wei Wang, Zhengrong Dai, Jie Li, Zhiyuan Xi, Kenan Jia, Yu Xing, Bingbing Li, Jiaqi Yan, Wensuo Jia

**Affiliations:** 1 College of Horticulture, China Agricultural University, Beijing, 100193 China; 2 College of International Education, Beijing University of Chemical Technology, Beijing, 100029 China; 3 College of Plant Science and Technology, Beijing University of Agriculture, Beijing, 102206 China

**Keywords:** Plant signalling, Plant molecular biology

## Abstract

An effector–reporter system is a powerful tool used to study cellular signal transduction, but this technique has been traditionally used in protoplasts. A similar system to study cellular signal transduction in fruits has not yet been established. In this study, we aimed to establish an effector–reporter system for strawberry fruit, a model nonclimacteric fruit. We first investigated the characteristics of transient gene expression in strawberry fruits and found marked variation in gene expression levels among individual fruits, and this variation has complicated the establishment of a technical system. To overcome this difficulty, we investigated a sampling strategy based on a statistical analysis of the activity pattern of four different reporters (GUS, GFP, FLuc, and RLuc) among individual fruits and combinations of pairs of reporters (GUS/GFP and RLuc/FLuc). Based on an optimized sampling strategy, we finally established a step-by step protocol for the effector/reporter assay. Using FaMYB10 and FaWRKY71 as the effectors and *GUS* driven by the *FaCHS* promoter as the reporter, we demonstrated that this effector/reporter system was practical and reliable. This effector/reporter technique will contribute to an in-depth exploration of the signaling mechanism for the regulation of strawberry fruit ripening.

## Introduction

Fruit ripening is a complex process that involves dramatic changes in various physiological and biochemical processes, such as color, sugar, acid, aroma, and texture-associated metabolisms^1–5^. Based on their physiological ripening characteristics, fleshy fruits can be categorized into two major groups, climacteric and nonclimacteric fruits^6–9^. Strawberry has increasingly become a model plant of nonclimacteric fruit. Studies have shown that the phytohormone abscisic acid (ABA) regulates fruit ripening in strawberry. In addition to phytohormones, the roles of various environmental stresses, such as light, heat, and osmotic stresses, in regulating strawberry fruit development and ripening have also been demonstrated^10–15^.

Molecular studies have identified genes implicated in the regulation of fruit development and ripening, and these mainly include structural genes, such as chalcone synthase (*CHS*), chalcone isomerase (*CHI*), dihydroflavonol 4-reductase (*DFR*), flavanone 3-hydroxylase (*F3H*), flavonoid 3′-hydroxylase (F3′H), anthocyanidin synthase (*ANS*), UDP glucose-flavonoid 3-O-glucosyl transferase (*UFGT*), sucrose phosphate synthetase, and sucrose synthetase (*SS*), which are directly involved in physiological and biochemical metabolism. In recent years, researchers have focused on transcription factors (TFs) that regulate fruit development and ripening. Many TFs, such as TFs belonging to the MYB^16^, NAC^17^, HLH^18^, and MADS^19^ families, have been implicated in fruit development and ripening. Studies have demonstrated that MYB10 regulates anthocyanin biosynthesis in various plant species and organs via anthocyanin biosynthesis genes, such as *CHS*, *F3H*, and *UFGT*^20,21^. However, less is known about the specific targets of TFs and the response of structural genes to the internal/external cues implicated in fruit development and ripening, and thus a detailed study of the cellular signal transduction pathways is needed.

Researchers have extensively studied the cellular signal transduction pathways in vegetative organs, but studies on fleshy fruits face many challenges. A major limiting factor in the study of cellular signaling in fruits is the shortage of related techniques and systems. The effector–reporter analysis technique has been used as a powerful tool to study the cellular signal transduction pathways underlying fruit development and ripening^22^. This technique is based on transient gene expression in plant cells^23–26^. Since the development of protoplast transient expression^27^, cellular signal transduction in protoplasts has been extensively studied with the aid of effector/reporter analyses. Earlier studies focused on responsive promoters, such as the ABA-responsive *Em* promoter in rice protoplasts^28^, GA-responsive amylase gene promoter in oat and barley aleurone protoplasts^29–31^, and the abiotic and biotic stress–responsive chalcone synthase (*CHS*) promoter^32–34^. In recent years, protoplast-based effector/reporter analysis has been increasingly employed to unravel different signaling cascades, including the two-component signaling circuit^35^ and the oxidative stress-activated MAPK cascade in Arabidopsis protoplasts, the auxin-triggered MAPK signaling cascade^36^ and the stress-induced CDPK cascade in maize protoplasts^37^ and, more recently^26^, the in vitro reconstitution of abscisic acid signaling in Arabidopsis. Protoplast-based effector/reporter systems have facilitated the rapid discovery of cellular signal transduction pathways in various biological processes, but some limitations exist^22^. First, it is not possible to isolate active protoplasts from each plant cell type or under all growth conditions. In addition, protoplasts cannot exhibit the biological processes of different cell types^22^. To date, the protoplast-based effector/reporter system has mainly been used in maize and Arabidopsis.

In addition to protoplast-based transient gene expression, the transient expression has been increasingly reported in the fruits of many plant species, and among these, strawberry maybe the plant species that have been most extensively studied^6,21,38–40^. Unlike that in protoplasts or tobacco leaves, transient gene expression in strawberry fruits is influenced by many factors, which results in a marked variation in gene expression levels among individual fruits^38^. The large variation in transient gene expression has become a major challenge to the establishment of an effector/reporter system in strawberry fruit because it would cause a large error that may cover up the real difference among samples. Thus, the development of a reasonable sampling strategy is needed to obtain reliable results. Therefore, in the current study, we first investigated the pattern of variation resulting from different reporters, and based on an analysis of the pattern of variation, we developed an optimized sampling strategy, i.e., the minimum sample size needed to obtain reliable results. A step-by-step protocol for the effector/reporter technical system is provided. This study contributes greatly to an in-depth exploration of the signaling mechanism for the regulation of strawberry fruit ripening.

## Results

### The pattern of sample variability

**Fig. 1:**
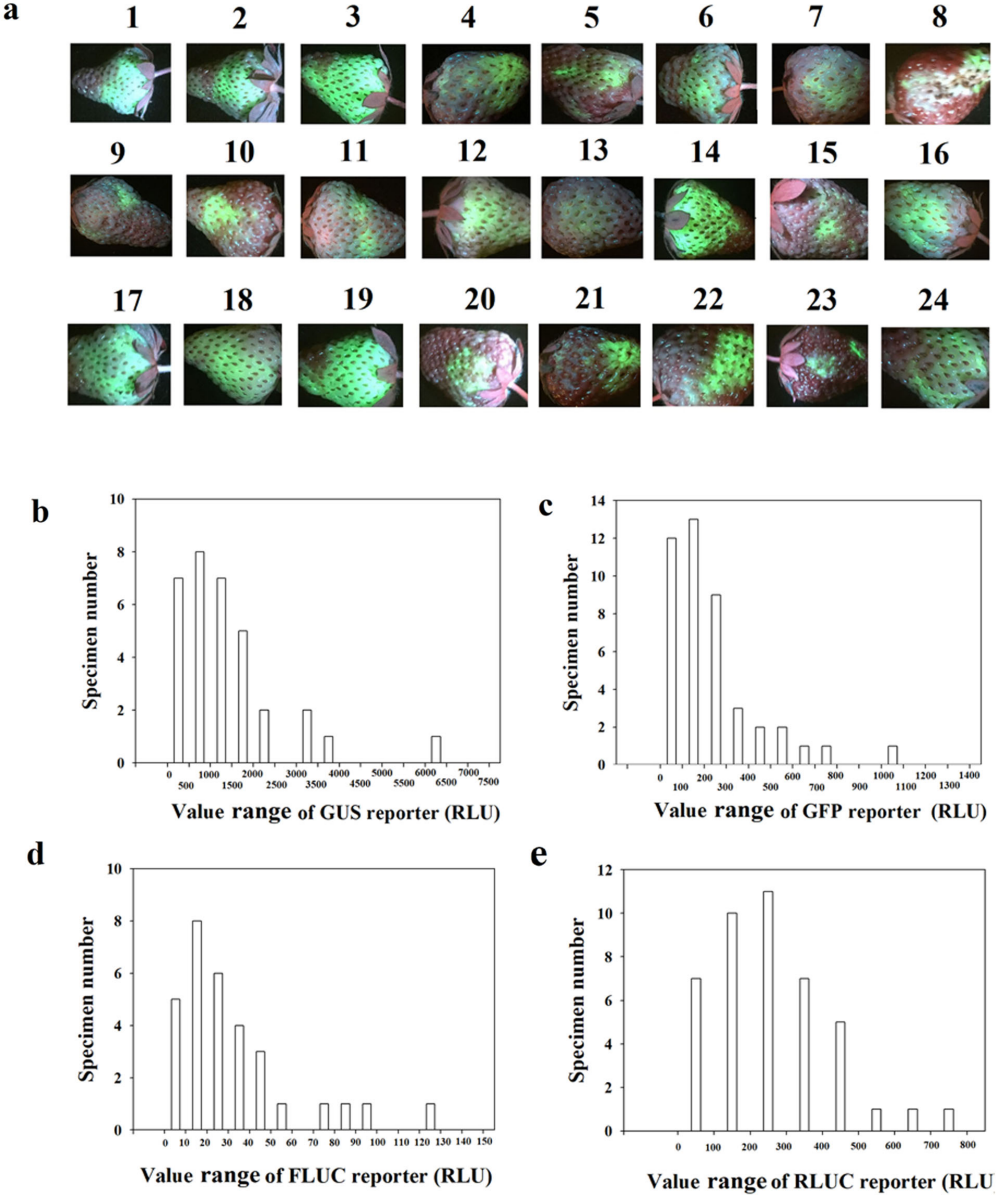
Data distribution of different reporters showing the variability of each reporter. **a** GFP fluorescence images showing the variability in the level of GFP protein among individual fruits with a sample size of 24. The number above each image denotes a random order in the sample. **b**–**e** Quantitative determination of the activity of different reporters. For each reporter, the data distribution is shown based on a specific sample group consisting of 30 individual fruits. Fruits at the LG stage were fully injected with Agrobacterium carrying the different single reporter constructs, and on 4 days after the injection, the reporter activity was measured. The vertical coordinate denotes the number of fruits with reporter activities within each corresponding region shown in the horizontal coordinate. The values were normalized by subtracting the background signal produced by the empty vector (i.e., the negative control). **b** GUS; **c** GFP; **d** FLuc; **d** RLuc; **e** GFP fluorescence image. More detailed statistical parameters are shown in Table 1

The transient expression of a gene, which is expressed as a relative fluorescent unit (RLU), shows marked variation among individual fruits (referred to as sample variability; SV). To characterize this variability in relation to the employment of different reporters, we examined four commonly used reporters, namely, GUS, GFP, FLUC, and RLUC. Figure 1a shows a general pattern of variability as indicated by an image of GFP fluorescence in a sample size of 24. Although strong GFP fluorescence could be clearly observed in some fruits, such as fruits 3, 14, and 19, the GFP fluorescence might be markedly weaker in others, such as fruits 4, 5, 7, and 21, and hardly even observed in some fruits, such as fruit 13. The specific pattern of the SV of different reporters is shown in Fig. 1b–e (the vertical coordinate denotes the number of specimens, and the horizontal coordinate denotes their values in the corresponding range). All four reporters exhibited high variability (0–1500 for GUS, Fig. 1b; 0–1500 for GFP, Fig. 1c; 0–150 for FLuc, Fig. 1d; and 0–800 for RLuc, Fig. 1e). This large SV implies that a relatively large number of fruits would be needed to satisfy the need for an analysis of the significance of the difference among different treatments in a study. The coefficient of variation (CV) and standard error of the mean (SEM) are two statistical parameters that are commonly used to assess the pattern of SV^41,42^. In the present study, we determined the percentage of the two parameters (percentage of CV (PCV) and percentage of SEM (PSEM) to assess the variability. Specifically, according to the formula: ${\mathrm{SD}} = \sqrt {\frac{1}{{N - 1}}\mathop {\sum}\limits_{i = 1}^N {(x_i - \bar x)^2} }$, where SD is the standard deviation, *x*_*i*_ is one sample value, *x̄* is the sample mean, and *N* is the sample size, and ${\rm{SE}} = \frac{{{\mathrm{SD}}}}{{\sqrt n }}$, where SE is the standard error, SD is the standard deviation, and *n* is the number of sample observations, the PCV and PSEM were determined as follows: PCV = SD/*x̄* × 100% and PSEM = SE/*x̄* × 100%.

**Fig. 2:**
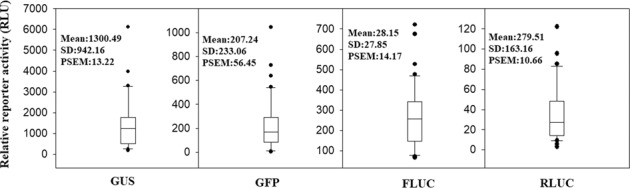
Box plot of different reporters showing the data pattern of the samples of each reporter. The box plot was prepared using the data shown in Fig. 1b (for GUS), 1c (for GFP), 1d (for FLUC), and 1e (for RLUC). *SD* standard deviation, *PSEM* percentage of standard error of the mean

To show the pattern of variation more intuitively, the data from Fig. 1b–e are shown in box plots in Fig. 2. The box plots clearly show that the data are quite dispersed and that many data points are even abnormal (i.e., the data exceed the upper and lower limits). These observations suggest that the development of a suitable sampling strategy is necessary to obtain reliable results. The development of a suitable sampling strategy aims to obtain a minimum PSEM. Because the PSEM might be caused by biological or technical replicates, to understand the precise contribution of biological and technical replicates to the PSEM value, we further examined the SV due to technical replicates. The variability due to the technical replicates was smaller than that due to the biological replicates. Moreover, the PSEM for GFP was markedly higher than those for the other reporters. The PSEM values for GUS, GFP, FLUC, and RLUC were 13.22%, 56.45%, 14.17%, and 7.44%, respectively, which indicated that GUS and RLUC could be higher-priority selections in the development of a technique for the assessment of cellular signal transduction (Table 1).

**Table 1:** Statistic parameters with an emphasis to show the effect of biological and technical replicates on the PSEM

	Mean	Median	SD	SE	95% Confidence	99% Confidence	PCV (%)	PSEM (%)
*Biological replicates*								
GUS	1300.49	1228.67	942.16	172.01	351.81	474.17	72.44	13.22
GFP	207.24	159.01	233.06	42.55	87.03	117.3	112.56	56.45
Fluc	35.87	28.15	27.85	5.09	10.41	14.02	77.65	14.17
Rluc	279.51	252.25	163.16	29.79	60.93	82.11	58.37	10.66
*Technical replicates*								
GUS	2638.47	2624.50	167.86	30.65	62.68	84.48	6.36	1.16
GFP	242.46	236.50	83.61	15.27	31.22	42.08	34.48	6.29
Fluc	62.13	62.50	22.97	4.19	8.58	11.56	36.97	6.75
Rluc	268.40	252.50	36.77	6.71	13.73	18.51	13.7	2.5

*SD* standard deviation, *SE* standard error, *PCV* Percentage of CV (coefficient of variation), *PSEM* Percentage of SEM (standard error of the mean). Statistic parameters were produced based on sample size 30 for each reporter

### Sampling analysis in relation to the sample size

**Fig. 3:**
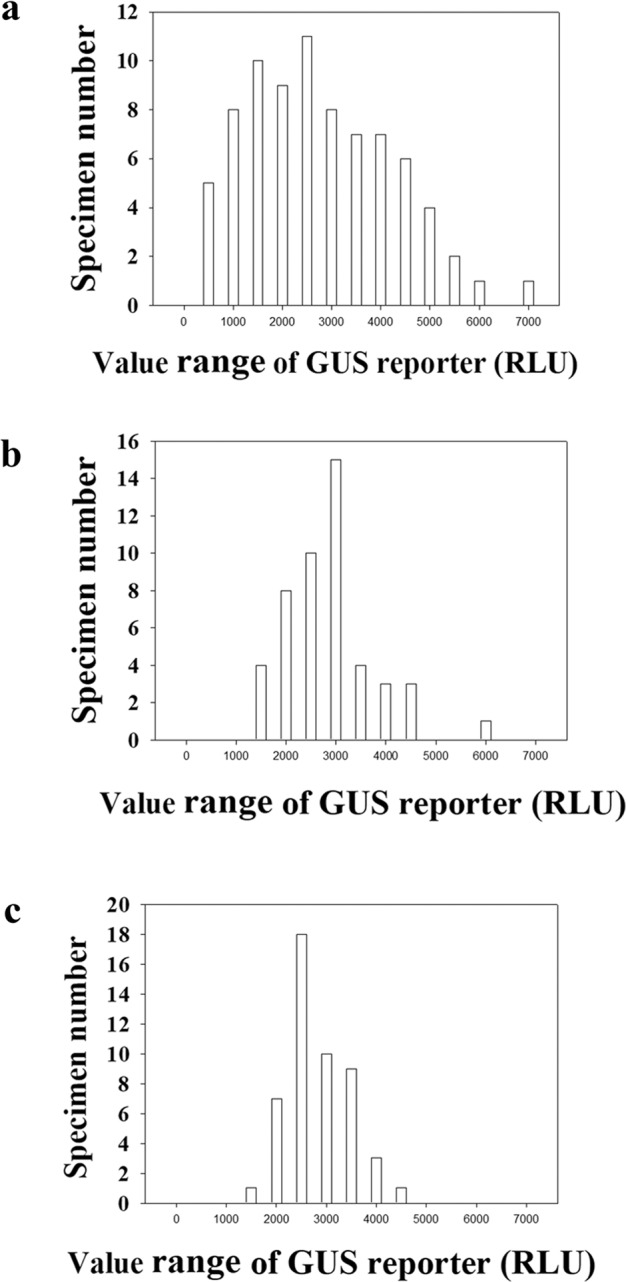
Data distribution of the GUS reporter showing the sampling effect on its data variability. Fruits at the LG stage were fully injected with the Agrobacterium carrying the *GUS* reporter construct, and 4 days after the injection, the reporter activity was measured. The vertical coordinate denotes the number of fruits with reporter activities within each corresponding region shown in the horizontal coordinate. The values were normalized by subtracting the background signal produced by the empty vector (i.e., the negative control). **a** The sample group consisted of 80 specimens, and each specimen represents an individual fruit; **b** the sample group consisted of 50 specimens, and each specimen represents a mixture of 3 individual fruits; **c** the sample group consisted of 50 specimens, and each specimen represents a mixture of 6 individual fruits. More detailed statistical parameters are shown in Table 2

Because PSEM is determined by both the sample size “*N*” and SE as described above, to determine the sample size for a given PSEM, we evaluated the relationship between sample size and PSEM with the GUS reporter (better choice). We first conducted the GUS analysis using a large sample size (80 individual fruits), and by random sampling, we then examined the change in PSEM with increases in the sample size. As shown in Fig. 3a, the data distribution obtained with a sample size of 80 was closer to a normal distribution than that obtained with a sample size of 30 (Fig. 1a). The calculated PSEM values for sample sizes of 5, 10, and 30 were 7.41%, 19.42%, and 11.42%, respectively (Table 2). Based on the assumption that a PSEM below 15% can be acceptable^41–46^, the sample size should exceed 30 to satisfy the needs of technical development. We further used mixed specimens with either three or six individual fruits to reduce the PSEM. For the mixed specimen with three fruits (Fig. 3b), we evaluated the PSEM for sample sizes of 5 and 10. The obtained PSEM values for sample sizes of 5 and 10 were 14.60% and 10.84%, respectively. For a mixed specimen with six fruits (Fig. 3c), we evaluated the PSEM values for a sample size of 5 and obtained a value of 10.33% (Table 2), which indicates that with the use of a mixed specimen, a sample size as low as 5 is sufficient to satisfy the test indicating a significant difference. Based on these findings, we used mixed specimens with a sample size of 5 for further experiments to study cellular signal transduction in strawberry fruits.

**Table 2:** Statistic parameters with an emphasis to show the effect of different sampling strategy on the PSEM

	Individual specimen			Mixed specimen of three fruits		Mixed specimen of six fruits
Number of fruits measured	81			50		49
Sample size	5	10	30	5	10	5
Sampling times	31	28	29	31	28	27
mean	27.41	19.43	11.42	14.60	10.84	10.33
median	25.524	18.12	11.33	14.86	10.36	10.86
SD	8.26	4.500	1.075	4.67	3.12	2.81
SE	1.48	0.900	0.21	0.85	0.59	0.54
95% Conf.	3.03	1.86	0.43	1.74	1.21	1.11
PSEM	5.41	9.56	3.800	11.94	11.15	14.56

*SD* standard deviation, *SE* standard error, *Conf.* confidence, *PCV* Percentage of CV (coefficient of variation), *PSEM* Percentage of SEM (standard error of the mean). A number of fruits measured, the number of fruits piratically measured for the reporter activity; sample size, the amount of data sampled each time from the practically measured data collection

**Fig. 4:**
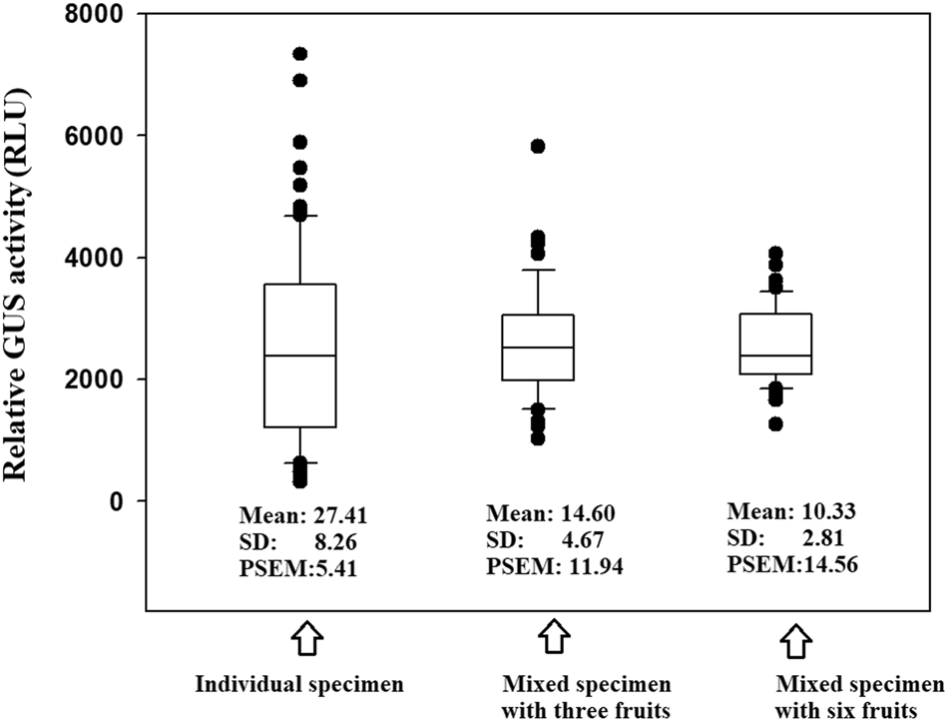
Box plot of different reporters showing the data pattern of the samples of each reporter. The box plot was prepared from the data shown in Fig. 3a (individual fruit sample), 3b (mixed sample of three fruits), and 3c (mixed sample of six fruits). SD standard deviation, PSEM percentage of the standard error of the mean

To show the pattern of variation more intuitively, the data from Fig. 3a–c are shown in box plots in Fig. 4. The box plots clearly show that the data are widely dispersed and that many data points are abnormal, as mentioned above. Notably, it can also be clearly seen that the data distribution obtained with a mixed specimen is higher than that obtained with an individual specimen and the greater the mixed number is, the more concentrated the data distribution becomes. These observations suggest that the adoption of a mixed specimen is a good strategy for obtaining reliable results.

### Comparison between single and double reporters

**Fig. 5:**
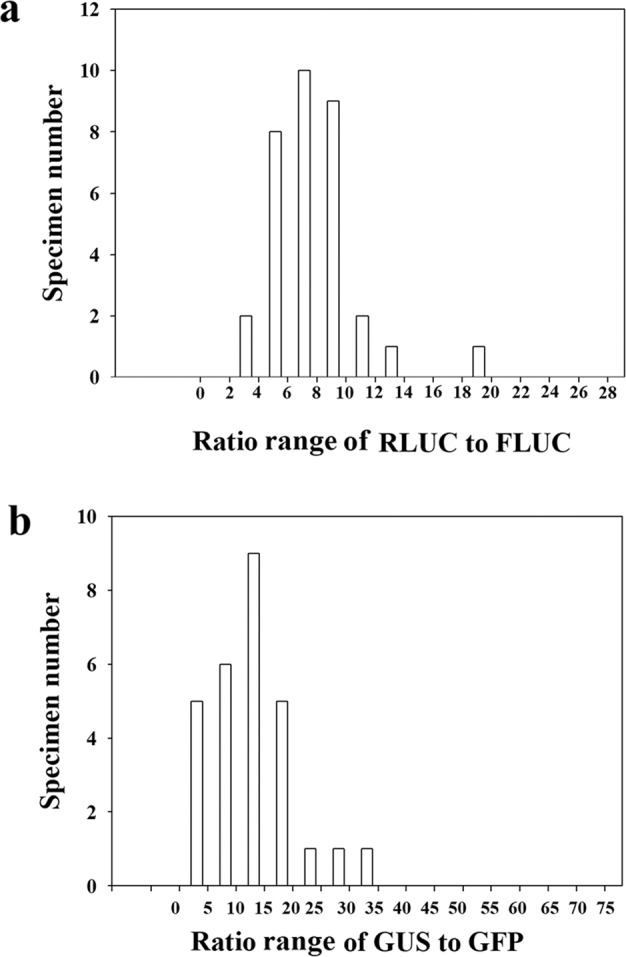
Data distribution showing the variability in the ratio of double reporters. Fruits at the LG stage were fully injected with Agrobacterium carrying a double reporter construct, and 4 days after the injection, the reporter activity was measured. The ratio of each individual fruit was calculated. The vertical coordinate denotes the number of fruits with ratio values within each corresponding region shown in the horizontal coordinate. The values were normalized by subtracting the background signal produced by the empty vector (i.e., the negative control). **a** Ratio of GUS to GFP with a sample group consisting of 30 individual fruits; **b** ratio of RLUC to FLUC with a sample group consisting of 33 individual fruits. More detailed statistical parameters are shown in Table 3

To characterize the effect of a ratio approach on the sampling SV, we first determined the ratios of different reporter combinations for approximately 30 individual fruits (GUS to GFP and RLuc to FLuc), and through random sampling, we evaluated the change in PSEM obtained with a change in the sample size. Figure 5 shows the pattern of variability for the different combinations. Compared with the single reporters (Fig. 1), the data with both reporters at different ratios were closer to the normal distribution for RLUC to FLUC (Fig. 5a) but not for GUS to GFP (Fig. 5b). As shown in Table 3, the ratio of PSEM for RLuc to FLuc was 7.71%, which was smaller than that obtained for the single reporters with the same sample size (10.6573% for RLuc and 14.17% for FLuc, sample size 30; Table 1). A PSEM value of 0.7393% (Table 3) was obtained with a sample size as low as 10 for the RLuc and FLuc combination. This value was markedly lower than the PSEM value obtained for a sample size of 30 with single reporters (Table 1).

**Table 3:** Statistic parameters with an emphasis to show the effect of biological replicates as well as the different sampling strategy on the PSEM for the ratio of RLUC to FLUC and GUS to GFP

	Practical measurement		Sampling from RLUC/FLUC	
	RLUC/FLUC	GUS/GFP	RLUC/FLUC	
Number of fruits examined	34	31	–	–
Sample size	–	–	5	10
Sampling times	–	–	30	30
Mean	7.71	11.97	14.77	10.73
Median	7.51	10.93	14.96	10.57
SD	3.01	8.19	7.82	4.23
SE	0.52	1.49	1.42	0.80
95% Conf.	1.07	3.05	2.92	1.64
99% Conf.	1.43	4.12	3.93	2.22
PCV	39.00	68.40	52.95	39.46
PSEM	6.79	12.48	9.66	7.45

*SD* standard deviation, *SE* standard error, *Conf.* confidence, *PCV* Percentage of CV (coefficient of variation), *PSEM* percentage of SEM (standard error of the mean). A number of fruits measured, the number of fruits piratically measured for the reporter activity; sample size, the amount of data sampled each time from the practically measured data collection

**Fig. 6:**
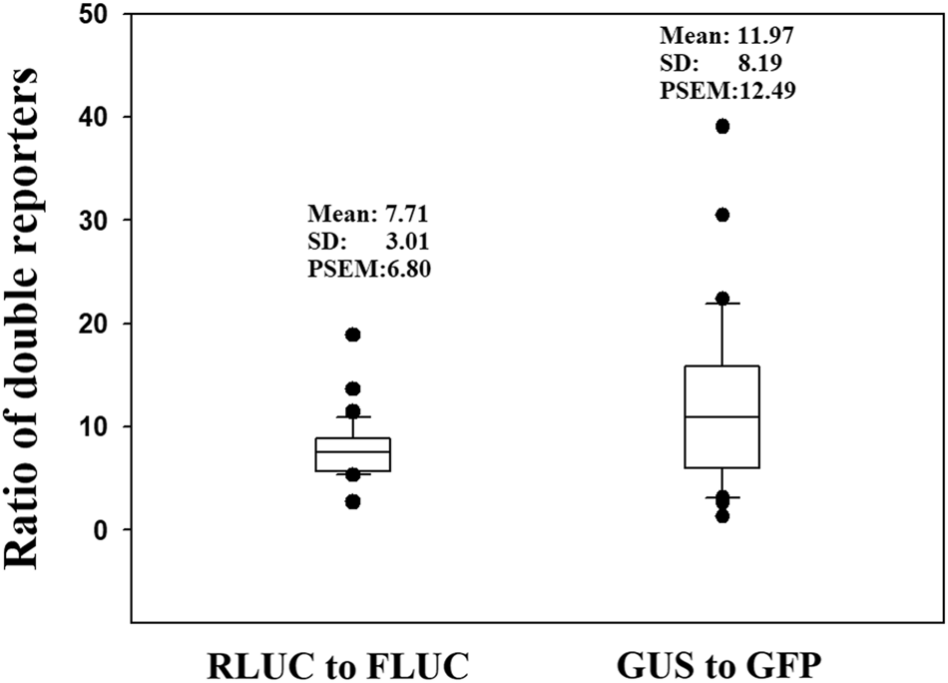
Box plot of different reporters showing the data pattern of the samples of each reporter. The box plot was prepared using the data shown in Fig. 5a (RLUC to FLUC) and 5b (GUS to GFP). SD standard deviation, PSEM percentage of the standard error of the mean

To show the pattern of data distribution more intuitionally, the data shown in Fig. 3a, b are presented in box plots in Fig. 6. Compared with that obtained for the ratio of GUS to GFP, the pattern found for the ratio of GUS to GFP was more concentrated, which indicated that the adoption of the ratio of RLUC to FLUC yielded relatively better results. These findings suggested that the use of ten individual fruits could satisfy the needs of studying cellular signal transduction in strawberry fruits.

### Application of the so-established technique to the study of cellular signal transduction

**Fig. 7:**
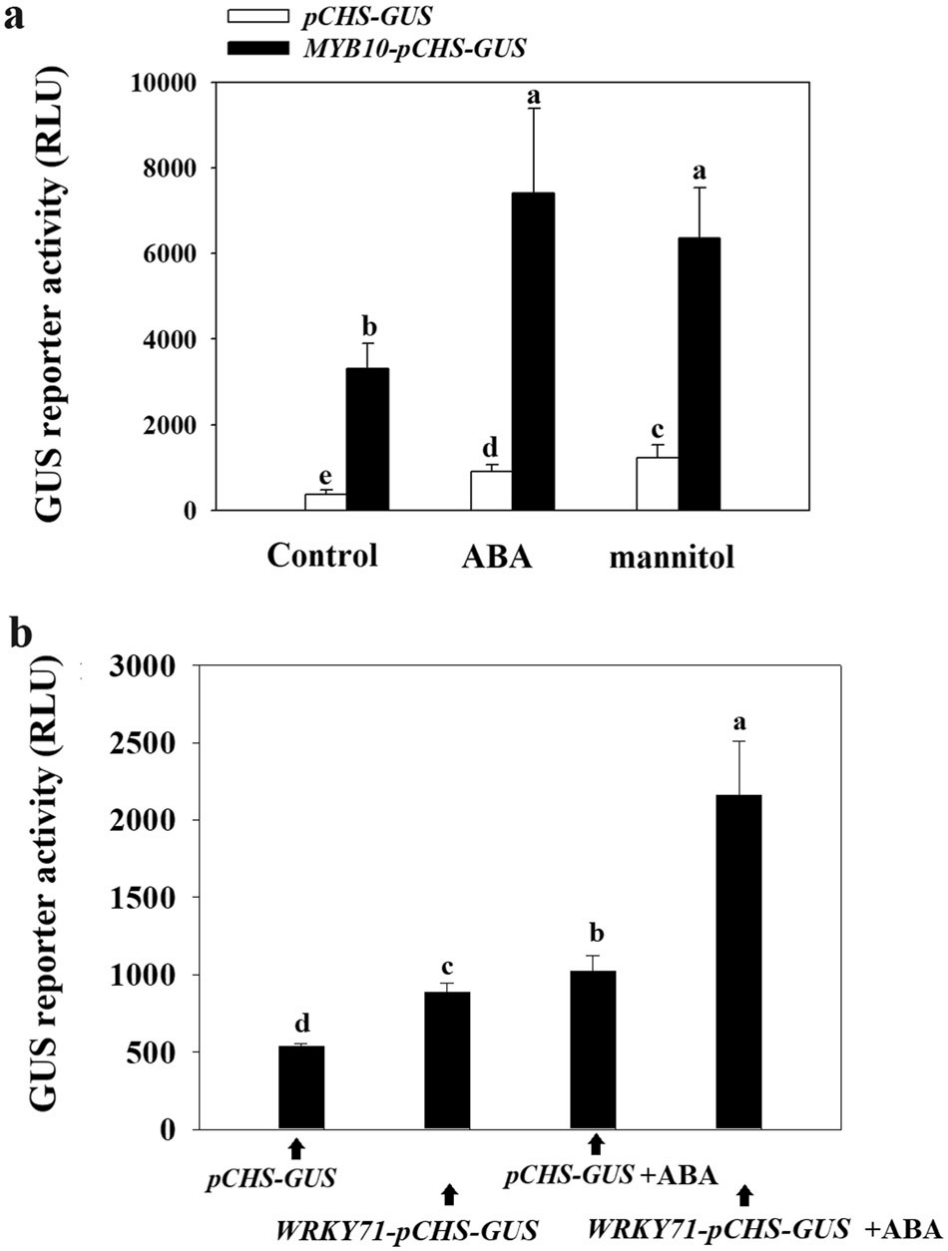
Analysis of signal transduction using the effector/reporter technical system in strawberry fruits. **a** *FaMYB10* driven by the 35S promoter (i.e., effector) and *GUS* is driven by the *FaCHS* promoter (i.e., reporter) were cloned into the same vector. Benihoppe fruits at the LG stage were fully injected with Agrobacterium carrying the “effector/reporter” constructor a single *GUS* reporter driven by the *FaCHS* promoter. Four days after the injection, the fruits were treated with 100 mM ABA or 700 mM mannitol for 5 h and then subjected to the measurement of GUS activity. **b** *FaWRKY71* driven by the 35S promoter (i.e., effector) and *GUS* is driven by the *FaCHS* promoter (i.e., reporter) were cloned into the same vector. Monterey fruits at the LG stage were fully injected with Agrobacterium carrying the “effector/reporter” constructor a single *GUS* reporter driven by the *FaCHS* promoter. Four days after the injection, the fruits were treated with 100 mM ABA. Each treatment included five specimens, and each specimen was a mixture of five individual fruits. The values were normalized by subtracting the background signal produced by the empty vector (i.e., the negative control). Different letters denote statistical significance with *P* < 0.05, and the same letter shows no significant differences among the groups (*P* ≥ 0.05)

The ratio method with a low SV was found to be relatively better, but this approach involves relatively high technical requirements and cost. Therefore, to test the application of the so-established technique, we evaluated the GUS reporter with a sample size of 5 using a mixture of five individual fruits as an independent sample (i.e., specimen), as described above. With *GUS* driven by the *FaCHS* promoter as the reporter and FaMYB10 as the effector, we investigated the response of the GUS reporter to *FaMYB10* and to ABA and mannitol treatment. The reporter activity significantly increased after either ABA or mannitol treatment (Fig. 7 and Table 4). The cotransformation of *FaMYB10* resulted in an increase in reporter activity of 7.33-fold compared with that obtained for the control without *FaMYB10*. To further demonstrate the reliability of this protocol, we conducted a comparative analysis of the transcriptome of strawberry fruit between the W and R stages and identified a TF, *FaWRKY71* (NCBI ID XM_004303826.2), that showed a great increase from the W to the R stage. With FaWRKY71 acting as an effector, we examined whether the *GU*S gene driven by the *FaCHS* promoter might be able to respond to ABA signaling. As shown in Fig. 7b, the addition of FaWRKY71 resulted in markedly higher GUS activity, particularly under ABA treatment, compared with that obtained without an effector. Compared with that obtained when only the GUS reporter was included, the activity of the GUS reporter was several-fold higher when both FaWRKY71 and ABA treatments were included in the system. Collectively, these results suggested that the so-established technical system can be easily applied to the study of cellular signal transduction in strawberry fruits. A step-by-step protocol of this technical system is provided in the “Materials and methods” section.

**Table 4:** Original data of each individual specimen (up panel) and their associated statistical parameters (low panel), with an emphasis to show the SD, SE, and PSEM as produced by the sampling strategy employed for the study of the signal transduction

	*pCHS-GUS*			*MYB10-pCHS-GUS*		
	Control	ABA	Mannitol	Control	ABA	Mannitol
Specimen 1	339	662	874	2485	5504	4650
Specimen 2	506	1084	1385	2996	8483	6646
Specimen 3	463	1024	936	3545	8693	7444
Specimen 4	271	853	1483	3621	9314	7352
Specimen 5	281	904	1476	3946	5068	5697
Mean	199.91	450.22	684.99	1683.59	4239.07	3237.31
SD	146.11	417.63	577.58	1606.48	3453.78	3001.20
SE	65.34	186.77	258.30	718.44	1544.58	1342.18
95% Conf.	181.42	518.54	717.15	1994.67	4288.33	3726.39
PSEM (%)	12.86	8.11	10.94	7.79	11.89	8.33

*SD* standard deviation, *SE* standard error, *Conf*. confidence, *PSEM* percentage of SEM (standard error of the mean). Specimen 1–5, a total of five specimens was measured for each treatment and each specimen was a mixture of 5 individual fruits

## Discussion

### Sampling strategy in relation to the exploitation of the effector–reporter system

A transient gene expression is a powerful tool used to study cellular signal transduction in plant cells^22^, but several limitations limit its use in fleshy fruits. In this study, we aimed to establish transient gene expression in strawberry fruits that will provide the foundation for the development of such a technical system. We previously found that transient gene expression in strawberry fruits can be influenced by various factors, such as the gene delivery method, fruit developmental stage, and environmental factors, and accordingly, conditional optimization is needed to perform transient gene expression in strawberry fruit^38^. The current study is based on the optimized conditions established in a previous study^38^. Due to the high variability in the gene expression levels (see Fig. 1 of the previous work), in the current study, we first further analyzed the statistical characteristics of the data distribution for different reporter samples. In the case of the GUS reporter, the difference between the minimum and maximum gene expression values was more than 50-fold (Fig. 1), which makes it difficult to study cellular signal transduction in strawberry fruits.

The study of cellular signal transduction essentially relies on analyses of the significance of the differences among samples (between samples with and without stimulation). In protoplast-based systems, a sample size of 3–5 (biological replicates) is usually used, and this sampling size provides an acceptable PSEM for assessing the significance of the differences (<15%)^23–25^. In the present study, we set an acceptable PSEM of approximately 10% and assessed the number of fruits needed for testing the significance of the differences. We propose that at least 30 individual fruits are needed to attain a significant difference (Table 1), which implies the need for several hundred fruits in a study with various treatments. Such a large number of fruits makes a study quite difficult to perform. Therefore, we evaluated the minimum sample size with which a system can be established to study cellular signal transduction in strawberry fruits.

The number of fruits required to develop a system is essentially determined by the statistical parameters SD and SE, which are associated with SV. Due to the difference in the absolute value of SE among samples, we adopted a relative SM (percentage of SM to the mean; designated as PSEM) in the present study. To investigate the relationship between PSEM and sample size, we adopted a random sampling strategy based on a relatively large sample group that was practically used to measure the reporter activity in each individual fruit. In random sampling, a small sample (5–30 specimens) was randomly chosen from a large sample group (50–80 specimens). Theoretically, the results from random sampling are identical to those obtained with a practically measured sample. Transient gene expression in strawberry fruits was manipulated via three major steps, including gene delivery, material grinding, and reporter activity analysis. To reduce the workload and cost involved in material grinding, we used mixed specimens. Non-mixed specimens with 30 individual fruits demonstrated a PSEM of 11.423% (Table 2), whereas mixed specimens (each specimen consisted of six fruits, and a sample size of 5 was adopted) with only five specimens produced a PSEM value of 10.333% (Table 2). Although the total number of fruits in both cases was similar, the use of mixed specimens reduced the workload and cost by five-sixths.

### Comparison of the ratio method versus the single reporter method

The ratio method has been traditionally thought to be better than the single reporter method. This approach adopts double reporters and uses the ratio of the two reporters as the output signal. The advantage of this approach is that its SD is relatively lower for a given group of samples, and thus, one can use relatively fewer samples to obtain reliable results. The disadvantage of this approach is that one needs to measure two reporters, and thus, two types of corresponding instruments might be needed; moreover, this approach is relatively more difficult to perform and costly. In the present study, we evaluated the ratio of two pairs of reporters, GUS to GFP and RLUC to FLUC. The use of RLUC and FLUC resulted in a lower PSEM (7.71%) compared with the values obtained with the four individual signal reporters (13.22% for GUS, 56.45% for GFP, 14.17% for FLUC, 10.65% for RLUC; a sample size of approximately 30, Table 1). Surprisingly, the combination of GUS and GFP did not result in a lower PSEM (11.97%). The green fluorescence (GFP), which was examined with a TBS-380 Fluorometer (Turner Biosystems), appeared to be unstable, as reflected by high PSEM (56.45%; Table 1). This finding implies that the use of two reporters in combination must be based on an accurate determination of both reporters, and an error derived from any single reporter will result in a larger error in their ratio.

The use of the double reporter method is better due to the low PSEM; however, there are some disadvantages related to its practical use. In addition to the cost and equipment involved, one major disadvantage is the difficulty involved in vector construction. In protoplasts, transient gene expression can be performed by cotransformation of different vectors, and the effector gene and the reporter gene are cloned separately into two different vectors. In contrast, the cotransformation of different vectors is impossible due to the large SV in strawberry fruits. To study cellular signal transduction, several genes need to be cloned into a single vector. In the case of a single reporter, it is easy to clone an effector gene into the reporter vector. In the ratio approach, at least three genes (two reporters plus at least one effector) need to be cloned into the same vector, which makes vector construction difficult (due to limited cleavage sites). These disadvantages of double reporters can be avoided by the use of a single reporter, which can result in an assessment of the significance of the differences with acceptable sample size. The use of mixed specimens with six individual fruits and sample sizes as low as 5 could result in a satisfactory PSEM (e.g., 10%). Collectively, the results showed that the ratio method was better than the single reporter method due to its smaller PSEM with given sample size, whereas both methods can be used, and for a specific researcher, the choice of method should depend on the available experimental conditions.

### Strawberry cultivar in relation to the exploitation of the effector–reporter system

In the current study, the effector–reporter system was established in *Fragaria* × *ananassa* Duch. Benihoppe. We previously tested the pattern of transient gene expression in different strawberry cultivars, including *Fragaria ananassa* Duch. “Benihoppe”, “Honeoye”, “Sweet Charlie”, “Albion”, and “Monterey”^38^, and found that all the cultivars showed a similar pattern of gene expression, which implies that the effector–reporter system can be well established in different strawberry cultivars. The sensitivity of the fruit response to external or internal stimuli might vary depending on the genetic background, which has raised the question of whether the effector–reporter system may be affected by the strawberry variety. It is well-known that the effector–reporter system based on maize or *Arabidopsis* protoplasts has been commonly used in many different plants species^23–25^, which implies that there is essentially no difference among cultivars in terms of the presence of a specific signaling cascade among different cultivars. Indeed, ABA has been demonstrated to play an important role in strawberry fruit ripening^10^, and there is no evidence indicating that ABA plays an important role in some strawberry cultivars but not in other cultivars.

FaCHS, a gene encoding chalcone synthase, controls color development^47^ and has been a gene of interest. The transcriptional regulation of CHS has been achieved by a complex of R2R3-MYB and basic helix–loop–helix (bHLH) TFs^48^. In strawberry, FaMYB10 regulates FaCHS^39,49^, and considering this regulatory role, we evaluated an effector–reporter system with FaMYB10 as the effector and GUS drove by the FaCHS promoter as the reporter to study cellular signal transduction in strawberry fruits. The highly sensitive response of the GUS reporter to the FaMYB10 effector as well as ABA and mannitol stimuli indicates the reliability and practicability of the so-established system. Apart from FaMYB10, to demonstrate the reliability of this protocol, we further examined whether FaWRKY71, a transcription factor that is greatly increased in gene expression during strawberry fruit ripening, might be able to serve as an effector to regulate *FaCHS* expression. Although the effector–reporter system was evaluated using the FaMYB10 and FaWRKY71 TFs, this does not mean that the effector–reporter system can only be applied to the analysis of an already known TF and its downstream target because the effector system is not limited to the combination of TFs/target genes. Collectively, the results show that the effector-reporter system can be applied to any effector–reporter combination in any strawberry cultivar.

### The exploitation of the effector/reporter system to explore the signaling mechanisms for fruit ripening

The effector/reporter system essentially enables the gene encoding a signal protein and the reporter gene driven by an output signal (i.e., promoter of the gene responding to the signal) to be coexpressed in cells. Thus, any signal proteins as well as transcription factors, such as receptors, protein kinases, and phosphatases, can act as effectors, whereas any gene responding to the signal can serve as the target gene (i.e., the output signal or the reporter gene driven by the promoter of the target gene), regardless of whether the effector activates or represses the reporter. Because this effector/reporter system was established in strawberry fruit, it is particularly applicable to research on strawberry fruit development and ripening. As fruit ripening involves marked changes in a series of biochemical metabolisms, such as color, sugar, acid, aroma, and cell walls, the genes encoding the key enzymes in these metabolic pathways are all interesting candidate reporters to research. In recent years, transcription factors have been increasingly studied for their involvement in fruit development and ripening, and thus, one common exploitation of the effector/reporter system might be the identification of the target gene. In addition, the system can also be used for the identification of *cis*-elements via an element deletion strategy. Compared with a stable transgene, the greatest advantage of the effector/reporter system is time savings: the system makes it possible to screen for a large number of candidate signals or target genes and not just demonstrate a regulatory relationship between a candidate signal and its target gene. The effector/reporter system could thus become a powerful tool for the in-depth exploration of the mechanisms involved in the regulation of strawberry fruit ripening.

## Materials and methods

### Plant materials

Octoploid strawberry plants (*Fragaria* × *ananassa* Duch., Benihoppe) were grown in a greenhouse at 18–28 °C and 75–90% humidity under an 8-h dark/16-h light cycle. The strawberry fruits were classified into six developmental stages as follows: small green fruit (SG), mid-sized green fruit (MG), large green fruit (LG), white fruit (W), turning fruit (T), and fully reddened fruit (FR). Fruits at the LG to W stages were used in the study.

### Reagent and buffers

Agar, casein tryptone, and yeast extract were derived from OXOID (UK). NaCl, MgCl_2_, EDTA, glycerin, Triton X-100, DTT, Tris-HCl (pH 8.0), 4-MUG, ethanol, NaH_2_PO_4_·2H_2_O, and Na_2_HPO_4_·2H_2_O were purchased from Sigma (USA). Antibiotics, including Amp, Kan, Sp, and rifampicin, were purchased from Sigma (USA). TransStart^®^ FastPfu PCR SuperMix (high-fidelity enzyme) was purchased from TransGen Biotech (China). Restriction endonuclease and T4 DNA ligase were purchased from NEB (USA). DH5a (large intestine) competent and EHA105 (agricultural rod) competent strains were purchased from TransGen Biotech Company. GV3101 (pSoup) chemically competent cells were purchased from ZOMANBIO (China). The TransDetect Double-Luciferase Reporter Assay Kit was purchased from TransGen Biotech (China).

The main buffer was prepared from four reagents (Luciferase Reaction Buffer, Luciferase Reaction Substrate (Lyophilized), Luciferase Reaction Buffer II, and Luciferase Reaction Substrate II (50×)) in the LUC test kit. To prepare Luciferase Reaction Buffer I, Luciferase Reaction Reagent Luciferase Reaction Substrate was completely dissolved in Luciferase Reaction Buffer (5 mL of Buffer + 1 vial of Substrate) and was stored in the dark. To prepare Luciferase Reaction Buffer II, Luciferase Reaction Reagent II and Luciferase Reaction Substrate II were mixed at a ratio of 1:50, and the reagent was stored in the dark after packaging.

### Vector construction

#### Construction of single reporters

The pCAMBIA1301 vector, which carries the *GUS* reporter gene driven by the cauliflower mosaic virus 35S promoter, was adopted to construct the GUS reporter. This vector was purchased from YouBio (China).

The pH7WG2D.1 vector, which carries the *eGFP* reporter gene driven by the *Agrobacterium rhizogenes* plasmid proID promoter, was adopted to construct the GFP reporter. This vector was purchased from BioVector NTCC Inc. The pGreenII 0800-LUC vector, which carries both FLuc and RLuc driven by the mosaic virus 35S promoter, was adopted to construct a single reporter (FLuc or RLuc) and was purchased from YouBio (China).

#### Construction of double reporters

The pH7WG2D.1 vector, which carries *eGFP* driven by the proID promoter, was used as the backbone to construct the GUS/GFP double reporter. The *GUS* gene was first amplified from pCAMBIA1301 using sense (5′-AAAAAGCAGGCTATGGTAGATCTGAGGGT-3′) and antisense (5′-AGAAAGCTGGGTTCACACGTGGTGGT-3′) primers. The PCR product was introduced into the lethal region of the *ccdB* gene of the pH7WG2D.1 vector using Gateway technology to obtain a GUS/GFP double reporter, which was designated the p35S::GUS-proID::GFP vector. The pGreenII 0800-LUC vector (purchased from YouBio), which carries the *RLUC* gene driven by the mosaic virus 35S promoter as well as the *FLUC* gene with multiple cloning sites, was used as the backbone to construct the RLuc/FLuc double reporter. The cauliflower mosaic virus 35S promoter was digested from pCAMBIA1301 and cloned between the *Hind*III and *Nco*I sites of the pGreenII 0800-LUC vector to obtain the RLuc/FLuc double reporter designated the p35S::RLUC-p35S::FLUC vector.

#### Construction of the effector and the reporter

The pCAMBIA1301 vector, which carries the *GUS* gene and a multiple cloning site in front of the *GUS* gene, was used as the backbone to construct the *CHS* reporter. The *CHS* promoter was amplified from the genome of *Fragaria* × *ananassa* Duch. (Benihoppe) using sense (5′-AAGCTTTTATGCTGATTTGATTATGTGT-3′) and antisense (5′- CCATGGTTTGATTTCTCAGAGAAGTGTC-3′) primers. The PCR products were cloned into the *Hind*III and *Nco*I sites of the pCAMBIA1301 vector to obtain the *CHS* reporter, which was designated the pCHS::GUS vector.

The pCHS::GUS vector was used as the backbone to construct the *FaMYB10* effector. The full-length coding sequence of *FaMYB10* was amplified from the cDNA of *Fragaria* × *ananassa* Duch. (Benihoppe) using sense (5′-GGATCCATGGAGGGTTATTTCGGTGT-3′) and antisense (5′-GAGCTCATTTTCTAATTGTAGAGTCTGTGG-3′) primers. The PCR products were cloned into pBI121 with *BamH*I and *Sac*I to fuse with the 35S promoter. The expression cassette was amplified using sense (5′-GTCGACTGAGACTTTTCAACAAAGG-3′) and antisense (5′-CCCGGGGATCTAGTAACATAGATGA-3′) primers, and the PCR product was cloned into the *Sal*I and *Sma*I sites of the pCHS::GUS vector to obtain the effector/reporter construct designated p35S::MYB10-pCHS::GUS.

The construction of the FaWRKY71 effector was based on the backbone of the pCHS::GUS vector. The full-length coding sequence of *FaWRKY71* was amplified from the cDNA of *Fragaria* *×* *ananassa* Duch (Monterey) with the following primer pairs: sense primer 5′-TCTAGAATGTCAAATGAAAAGAAAAGCCCT-3′ and antisense primer 5′-GAGCTCTCATGGCTCCTCCAGCTTGTGACT-3′. The PCR products were then introduced into pBI121 with *XbaI* and *SacI* to fuse with the 35S promoter. The expression cassette was amplified with the sense primer 5′-GTCGACTGAGACTTTTCAACAAAGG-3′ and antisense primer 5′- CCCGGGGATCTAGTAACATAGATGA-3′, and the PCR product was then cloned into the *SalI* and *SmaI* sites of the pCHS::GUS vector, which yielded the effector/reporter construct, designated p35S::WRKY71-pCHS::GUS.

### Sampling strategy

The relationship between the PSEM and sample size was established by both practical measurements and random sampling. We first measured the reporter activity of each fruit using a relatively large sample size (e.g., a sample size of 81 for non-mixed specimens, a sample size of 50 for mixed specimens of three fruits and a sample size of 49 for mixed specimens of six fruits for the GUS reporter). We evaluated the PSEM for sample sizes of 5, 10, and 30 with non-mixed specimens, for sample sizes of 5 and 10 with mixed specimens of three fruits, and for a sample size of 5 with mixed specimens of six fruits. Random sampling was performed 27–31 times to ensure a sampling PSEM below 15% using the Microsoft Excel ‘RAND function’.

### Statistical analysis

The biological or technical replicates were set from 5 to 80 according to the different aims of the experiments as described above. The evaluation of statistical parameters as well as the analysis of the significance of the differences were conducted using SigmaPlot (Systat Software, Inc.) for Windows.

### GFP fluorescence image analysis

The GFP fluorescence in images was analyzed using an independently developed apparatus for analyzing and imaging objects with a surface area of up to 100 cm^2^ that comprises an argon laser, a 488-nm excitation filter, and a 507-nm emission filter.

### GUS and GFP analyses

Approximately, 10 µL of the protein extract was mixed with 100 µL of reaction buffer (10 mM Tris-HCl (pH 8.0), 2 mM MgCl_2_, and 1 mM 4-MUG) in a 2-mL vial and incubated at 37 °C for 60 min, and 900 µL of 0.2 M Na_2_CO_3_ was then added to stop the reaction. The reaction mixture was subsequently transferred to a colorimetric cup adapted to a TBS-380 mini fluorometer with two excitation modes (UV; 365–395 nm and blue; 465–485 nm), which produced two emission spectra (440–470 nm for UV and 515–575 nm for blue). GUS activity was measured with the excitation set to the UV mode and with an emission wavelength of 365 nm. This activity is expressed as the relative fluorescence unit (RLU). For GFP measurement, the protein extract was transferred into a colorimetric cup, the excitation was set to blue, and the emission wavelength was 465 nm.

### RLuc (*Renilla luciferase*) and FLuc (*Firefly luciferase*) analyses

RLuc and FLuc were analyzed using the Double-Luciferase Reporter Assay Kit following the manufacturer’s protocol. Luciferase Reaction Reagent I (100 μL) was equilibrated to room temperature and thoroughly mixed with 20 μL of the protein extract. The reaction mixture was transferred into a 1.5-mL vial and measured immediately with a TD20-20 Luminometer. The FLuc activity was expressed as the RLU. Furthermore, 100 μL of Luciferase Reaction Reagent II was added to the reaction mixture and measured immediately with a TD20-20 Luminometer. The RLuc activity was expressed as the RLU.

### A step-by-step protocol for the study of cellular signal transduction with single or double reporters

#### Step 1. Preparation of fruits

We used octoploid strawberry, *Fragaria* × *ananassa* Duch. (Benihoppe), in this study. The plants were grown in a greenhouse, and the fruits were divided into six developmental stages as described above. Fruits at the large green to white stages were chosen for the study, and fruits at a uniform stage were used for each experiment. The selection of fruits was based on the achene color (breaking stage). The number of fruits per experiment was decided as described below. The fruits were harvested by cutting from the petiole and were immediately transported to the laboratory for infection.

#### Step 2. Sampling design

Each fruit was individually infected, and five infected fruits were pooled for grinding to obtain mixed specimens. At least five biological replicates were maintained; thus, for each treatment, 25 fruits were maintained. Fruits infected with an empty vector (no effector/reporter) served as the control.

#### Step 3. Preparation of the bacterium carrying the target vector

1. Bacterial culture
  Liquid culture medium and antibiotics were used based on the target vector. Kanamycin was used for pCAMBIA1301, pCHS::GUS, and p35S::RLUC-p35S::FLUC, and streptomycin was used for pH7WG2D.1. The agrobacterium strain EHA105 carrying the target vector was first cultured in 20 mL of the medium at 200 rpm and 28 °C until the OD600 reached 0.6. This culture was further grown under the same conditions until the OD600 reached 0.6 in a large volume, which was determined by the number of fruits required for the infection. Normally, each individual fruit needs 1–3 mL.
2. Strain activation
  The bacterium was collected by centrifugation at 4000 rpm for 10 min and resuspended in activation medium (10 mM MgCl_2_, 200 μM acetosyringone, and 10 mM MES, pH 5.6) to obtain an OD600 of 0.5–0.8. The culture obtained after 3–4 h of incubation at 28 °C was used for infection. The activation medium cannot be stored, and the fruits should be prepared in advance.
3. Fruit infection

Agrobacterium suspension was injected into the fruits using a 1-mL syringe. The needle tip was inserted into the fruit center from either the top or the bottom, and the suspension was slowly and evenly injected into the fruits until the whole fruit was fully infiltrated. If 1 mL was not enough to achieve full infiltration, the fruits were injected more than once. The infiltrated fruits were incubated in the dark at 20–25 °C with 90% humidity for 4 days. The infected fruits were either used immediately for the different experiments or frozen in liquid nitrogen and maintained at −80 °C for further use. Fruits infected with the corresponding empty vector served as the negative control, and the value of the reporter signal was normalized by subtracting the background signal produced by the empty vector.

#### Step 4. Fruit treatment for studying cellular signal transduction

To study the response of the reporter to different stimuli, the fruits were treated with the corresponding stimuli. In the present study, each fruit was cut into four equal parts and incubated with 100 or 700 mM mannitol for 5 h at room temperature. If treatment is necessary in some studies, the infected fruits can be treated as described in step 3.

#### Step 5. Protein extraction for reporter analysis

The fruits were frozen in liquid nitrogen. Five fruits were mixed and ground into a fine powder with a mortar and pestle. For each assay, 100 mg of the fine powder was weighed and added to a 2.0-mL vial containing 500 µL of extraction buffer (1 mM EDTA, 10% glycerol, 0.5% Triton X-100, 1 mM DTT, and 100 mM Na_2_HPO_4_–NaH_2_PO_4_; pH 7.8). This mixture was incubated in a shaking incubator at 4 °C for 1 h and centrifuged for 10 min at 13000 rpm and 4 °C. The supernatant was used to analyze the reporter activity. If a single reporter is to be adopted, we recommend GUS because according to our experience, the GUS reporter is reliable and relatively easy to measure (using a TBS-380 mini fluorometer or any other fluorometer). If double reporters are to be adopted, we recommend FLUC/RLUC as the reporter because the vector and the assay kit are commercially available.

## References

[1] Coombe BG (1976). The development of fleshy fruits. Annu. Rev. Plant Physiol..

[2] Brady CJ (1987). Fruit ripening. Annu. Rev. Plant Physiol..

[3] Giovannoni J (2001). Molecular biology of fruit maturation and ripening. Annu. Rev. Plant Physiol..

[4] Seymour GB, Østergaard L, Chapman NH, Knapp S, Martin C (2013). Fruit development and ripening. Annu. Rev. Plant Biol..

[5] Giovannoni J, Nguyen C, Ampofo B, Zhong S, Fei Z (2017). The epigenome and transcriptional dynamics of fruit ripening. Annu Rev. Plant Biol..

[6] Guidarelli M, Baraldi E (2015). Transient transformation meets gene function discovery: the strawberry fruit case. Front. Plant Sci..

[7] Cherian S, Figueroa CR, Nair H (2014). ‘Movers and shakers’ in the regulation of fruit ripening: a cross-dissection of climacteric versus non-climacteric fruit. J. Exp. Bot..

[8] Moya-León MA, Mattus-Araya E, Herrera R (2019). Molecular events occurring during softening of strawberry fruit. Front. Plant Sci..

[9] Haugeneder A (2018). Answering biological questions by analysis of the strawberry metabolome. Metabolomics.

[10] Jia HF (2011). Abscisic acid plays an important role in the regulation of strawberry fruit ripening. Plant Physiol..

[11] Han Y (2015). Sucrose nonfermenting1-related protein kinase2. 6, an ortholog of OPEN STOMATA1, is a negative regulator of strawberry fruit development and ripening. Plant Physiol..

[12] Liao X (2018). Interlinked regulatory loops of ABA catabolism and biosynthesis coordinate fruit growth and ripening in woodland strawberry. Proc. Natl Acad. Sci. USA.

[13] Chen J, Mao L, Lu W, Ying T, Luo Z (2016). Transcriptome profiling of postharvest strawberry fruit in response to exogenous auxin and abscisic acid. Planta.

[14] Medina-Puche L (2016). Extensive transcriptomic studies on the roles played by abscisic acid and auxins in the development and ripening of strawberry fruits. Funct. Integr. Genomics.

[15] Xie YG (2020). Transcription factor FvTCP9 promotes strawberry fruit ripening by regulating the biosynthesis of abscisic acid and anthocyanins. Plant Physiol. Biochem..

[16] Medina-Puche L (2015). An R2R3-MYB transcription factor regulates eugenol production in ripe strawberry fruit receptacles. Plant Physiol..

[17] Carrasco-Orellana C (2018). Characterization of a ripening-related transcription factor FcNAC1 from Fragaria chiloensis fruit. Sci. Rep..

[18] Li Y (2020). FvbHLH9 functions as a positive regulator of anthocyanin biosynthesis by forming a HY5–bHLH9 transcription complex in strawberry fruits. Plant Cell Physiol..

[19] Vallarino JG (2020). Characterizing the involvement of FaMADS9 in the regulation of strawberry fruit receptacle development. Plant Biotechnol. J..

[20] Rahim MA, Busatto N, Trainotti L (2014). Regulation of anthocyanin biosynthesis in peach fruits. Planta.

[21] Medina-Puche L (2014). MYB10 plays a major role in the regulation of flavonoid/phenylpropanoid metabolism during ripening of Fragaria× ananassa fruits. J. Exp. Bot..

[22] Sheen J (2001). Signal transduction in maize and Arabidopsis mesophyll protoplasts. Plant Physiol..

[23] Yoo SD, Cho YH, Sheen J (2007). Arabidopsis mesophyll protoplasts: a versatile cell system for transient gene expression analysis. Nat. Protoc..

[24] Niu Y, Sheen J (2012). Transient expression assays for quantifying signaling output. Methods Mol. Biol..

[25] He P, Shan L, Sheen J (2007). The use of protoplasts to study innate immune responses. Methods Mol. Biol..

[26] Fujii H (2009). In vitro reconstitution of an abscisic acid signaling pathway. Nature.

[27] Cocking EC (1960). A method for the isolation of plant protoplasts and vacuoles. Nature.

[28] Marcotte WR, Bayley CC, Quatrano RS (1988). Regulation of a wheat promoter by abscisic acid in rice protoplasts. Nature.

[29] Huttly AK, Baulcombe DC (1989). A wheat α‐Amy2 promoter is regulated by gibberellin in transformed oat aleurone protoplasts. EMBO J..

[30] Gopalakrishnan B, Sonthayanon B, Rahmatullah R, Muthukrishnan S (1991). Barley aleurone layer cell protoplasts as a transient expression system. Plant Mol. Biol..

[31] Jacobsen JV, Close TJ (1991). Control of transient expression of chimaeric genes by gibberellic acid and abscisic acid in protoplasts prepared from mature barley aleurone layers. Plant Mol. Biol..

[32] Lipphardt S, Brettschneider R, Kreuzaler F, Schell J, Dangl JL (1988). UV‐inducible transient expression in parsley protoplasts identifies regulatory cis‐elements of a chimeric Antirrhinum majus chalcone synthase gene. EMBO J..

[33] Van de Löcht U, Meier I, Hahlbrock K, Somssich IE (1990). A 125 bp promoter fragment is sufficient for strong elicitor‐mediated gene activation in parsley. EMBO J..

[34] Loake GJ (1991). Phenylpropanoid pathway intermediates regulate transient expression of a chalcone synthase gene promoter. Plant Cell.

[35] Hwang I, Sheen J (2001). Two-component circuitry in Arabidopsis cytokinin signal transduction. Nature.

[36] Kovtun Y, Chiu WL, Zeng W, Sheen J (1998). Suppression of auxin signal transduction by a MAPK cascade in higher plants. Nature.

[37] Sheen J (1996). Ca2+-dependent protein kinases and stress signal transduction in plants. Science.

[38] Zhao Y (2019). Optimization and standardization of transient expression assays for gene functional analyses in strawberry fruits. Hort. Res..

[39] Wei L (2018). FaMYB44. 2, a transcriptional repressor, negatively regulates sucrose accumulation in strawberry receptacles through interplay with FaMYB10. J. Exp. Bot..

[40] Agius F, Amaya I, Botella MA, Valpuesta V (2005). Functional analysis of homologous and heterologous promoters in strawberry fruits using transient expression. J. Exp. Bot..

[41] Barde MP, Barde PJ (2012). What to use to express the variability of data: standard deviation or standard error of mean?. Perspect. Clin. Res..

[42] Nagele P (2003). Misuse of standard error of the mean (SEM) when reporting variability of a sample. A critical evaluation of four anaesthesia journals. Br. J. Anaesth..

[43] Doane JF, Mukerji MK, Olfert O (2000). Sampling distribution and sequential sampling for subterranean stages of orange wheat blossom midge, *Sitodiplosis mosellana* (Géhin) (Diptera: Cecidomyiidae) in spring wheat. Crop Prot..

[44] Smith SG (2011). Probability sampling of stony coral populations in the Florida Keys. Environ. Monit. Assess..

[45] Curran-Everett D (2008). Explorations in statistics: standard deviations and standard errors. Adv. Physiol. Educ..

[46] Larson MJ, Carbine KA (2017). Sample size calculations in human electrophysiology (EEG and ERP) studies: a systematic review and recommendations for increased rigor. Int. J. Psychophysiol..

[47] Yonekura-Sakakibara K, Higashi Y, Nakabayashi R (2019). The origin and evolution of plant flavonoid metabolism. Front. Plant Sci..

[48] Hartmann U, Sagasser M, Mehrtens F, Stracke R, Weisshaar B (2005). Differential combinatorial interactions of cis-acting elements recognized by R2R3-MYB, BZIP, and BHLH factors control light-responsive and tissue-specific activation of phenylpropanoid biosynthesis genes. Plant Mol. Biol..

[49] Laitinen RA, Ainasoja M, Broholm SK, Teeri TH, Elomaa P (2008). Identification of target genes for a MYB-type anthocyanin regulator in Gerbera hybrida. J. Exp. Bot..

